# A route for a strong increase of critical current in nanostrained iron-based superconductors

**DOI:** 10.1038/ncomms13036

**Published:** 2016-10-06

**Authors:** Toshinori Ozaki, Lijun Wu, Cheng Zhang, Jan Jaroszynski, Weidong Si, Juan Zhou, Yimei Zhu, Qiang Li

**Affiliations:** 1 Condensed Matter Physics and Materials Science Department, Brookhaven National Laboratory, Upton, New York 11973, USA; 2 Department of Nanotechnology for Sustainable Energy, Kwansei Gakuin University, 2-1 Gakuen, Sanda, Hyogo 669-1337, Japan; 3 National High Magnetic Field Laboratory, Florida State University, 1800 E. Paul Dirac Drive, Tallahassee, Florida 32310, USA

## Abstract

The critical temperature *T*_c_ and the critical current density *J*_c_ determine the limits to large-scale superconductor applications. Superconductivity emerges at *T*_c_. The practical current-carrying capability, measured by *J*_c_, is the ability of defects in superconductors to pin the magnetic vortices, and that may reduce *T*_c_. Simultaneous increase of *T*_c_ and *J*_c_ in superconductors is desirable but very difficult to realize. Here we demonstrate a route to raise both *T*_c_ and *J*_c_ together in iron-based superconductors. By using low-energy proton irradiation, we create cascade defects in FeSe_0.5_Te_0.5_ films. *T*_c_ is enhanced due to the nanoscale compressive strain and proximity effect, whereas *J*_c_ is doubled under zero field at 4.2 K through strong vortex pinning by the cascade defects and surrounding nanoscale strain. At 12 K and above 15 T, one order of magnitude of *J*_c_ enhancement is achieved in both parallel and perpendicular magnetic fields to the film surface.

## Abstract (web summary)

Simultaneous increase of critical temperature and critical current in superconductors is desirable for application purpose, but very difficult to realize. Here, Ozaki *et al*. report a simultaneous enhancement of *T*_c_ and *J*_c_ in FeSe_0.5_Te_0.5_ films with cascade defects produced by low-energy proton irradiation.

## Graphical abstract

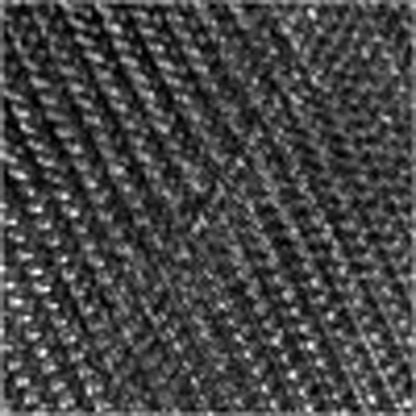

Superconductor's lossless current flow enables the design of highly dense and compact equipment, and hence offers powerful opportunities for increasing the capacity and the efficiency of power grids^1^,^2^. Superconducting coils use magnetic fields to store energy with near zero energy loss and can power drive rotary machines. Three properties of superconductors important for energy applications are superconducting transition temperature (also known as critical temperature) *T*_c_, upper critical field, and electrical current-carrying capacity. They arise from different aspects of the superconducting states. *T*_c_ is directly related to the mechanism of superconductivity. The superconducting state emerges at *T*_c_ from interactions between electrons that form the Cooper pairs. The upper critical field, *H*_c2_, is the highest magnetic field at which superconductivity is finally suppressed. In contrast to *T*_c_ and *H*_c2_, the current-carrying capacity, measured by the practical critical current density *J*_c_, is governed by the vortex pinning strength, which is determined by the ability of defects in superconducting materials to pin the vortices carrying magnetic flux. Defects create local regions with depressed pairing potential, which can pin the vortices. By doing so, unfortunately, defects tend to preclude the Cooper pair formation and hence drive down the *T*_c_ of cuprates and iron-based superconductors with short coherence lengths. It is a very difficult and challenging task to raise *T*_c_ and *J*_c_ simultaneously by means of introducing defects in the same cuprates and iron-based superconducting materials. Tremendous efforts have been made so far to optimize the defects landscape, to enhance *J*_c_, while keeping the degradation of *T*_c_ to a minimum in cuprates and iron-based superconductors. Indeed, great improvements in *J*_c_ have been obtained in cuprate and in iron-based superconducting films as well, by introducing artificially designed defects such as precipitate and columnar defects, but at the cost of *T*_c_ degradation^3^,^4^,^5^,^6^,^7^,^8^,^9^,^10^.

Iron-based superconductors^11^ have attracted much attention in potential high-field applications due to their relatively high *T*_c_, high *H*_c2_, and small anisotropy *γ*^12^,^13^. Recent reports show that high-quality epitaxial films have been grown on single crystal substrates, mostly used for exploring the fundamental properties of iron-based superconductors^8^,^9^,^10^,^14^,^15^,^16^,^17^,^18^. We have reported very high-*J*_c_ FeSe_0.5_Te_0.5_ (FST) films with CeO_2_ buffer layer on single crystals and a metal substrate of coated conductors^16^. We found that further *J*_c_ improvement by controlled growth of pinning defects, in the iron-based superconducting films we processed, is becoming increasingly difficult. This motivated us to look for alternative routes. Ion irradiation is a well-established method for artificially introducing various defects into superconducting materials in a fairly predictable and controllable manner by choosing appropriate ion species and energy^3^,^19^,^20^,^21^,^22^. Transmission electron microscopy (TEM) cross-sectional images along the ion traces in 230 MeV Au-irradiated Bi_2_Sr_2_CaCu_2_O_*x*_ single crystals showed several types of morphology of defects from parallel columnar defects to disordered cascade defects, as the ion energy is decreased in the crystals^22^. It is also well known that light ion irradiations produce smaller damaged volume fractions in the target materials than heavy ion irradiation and requires a much higher dosage to produce enough defects for flux pinning because of the significantly large ionization loss. These defects produced by ion irradiation would account for the depression of *T*_c_ in YBa_2_Cu_3_O_*y*_ (YBCO), which is proportional to the average number of defects^23^. Recently, a low-energy ion irradiation has been revisited and suggested as a practically feasible approach to improve flux pinning in YBCO^24^,^25^,^26^. In single crystals of iron-based superconductors, it has been found that ion irradiations, as a whole, improve *J*_c_ and its *J*_c_ enhancements persist up to much higher fluencies than in cuprate superconductors, although *T*_c_ is suppressed with increasing irradiation doses^27^,^28^,^29^. In contrast, the irradiated iron-based superconducting films have not shown as positive effects as found in single crystals^30^,^31^. It is clear that the key to the enhancement of *J*_c_ in superconductors is in the design of defect landscape that must be tailored to a superconductors' chemistry, crystal structure, and materials' geometric aspect.

Here we show a strategy on nanostructure control in iron-chalcogenide superconducting FST films with low-energy proton irradiation. Through extensive TEM characterization, we found nanoscale strain modulations in FST films irradiated with 1 × 10^15^ p cm^–2^ dose of 190 keV proton. Enhanced 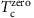 from 18.0 to 18.5 K is observed in the irradiated films due to the nanoscale compressive strain and proximity effect. The low-energy proton irradiation also yields self-field *J*_c_ enhancement from 0.9 MA cm^–2^ up to 1.4 MA cm^–2^ at 4.2 K. The cascade defects and nanoscale strain produced by low-energy proton irradiation are found to provide strong vortex pinning, resulting in remarkable enhancements of *J*_c_ at both zero field and high magnetic field in all temperatures, and high maximum pinning force *F*_p,max_ of ∼120 GN m^–3^ for *H*//*ab* at 27 T and 4.2 K.

## Results

### Transition temperature and microstructural analysis

**Figure 1:**
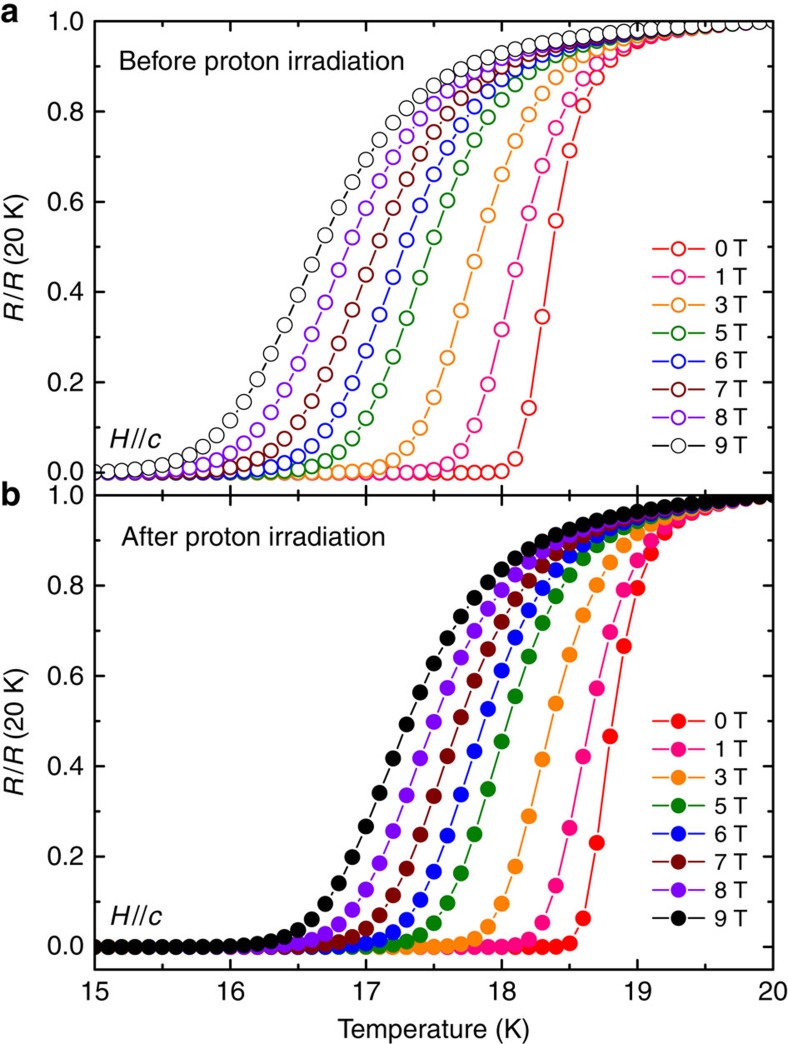
Temperature dependence of normalized resistivity. *ρ*(*T*)/*ρ*(20 K) at 0–9 T//*c* for the FST film before (**a**) and after (**b**) 190 keV proton irradiation with 1 × 10^15^ p cm^−2^ dose.

Figure 1a,b show the temperature dependence of the normalized resistivity up to 9 T with *H*//*c* for the FST film before and after 190 keV proton irradiation at 1 × 10^15^ p cm^−2^ fluence, respectively. We determine the 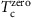 and the irreversibility field (*H*_irr_) using 0.01*ρ*_n_ criteria and *H*_c2_ using 0.9 *ρ*_n_ criteria with *ρ*_n_=*ρ*(20 K). As previously reported^16^, the CeO_2_-buffered FST film (pristine film) exhibits enhanced 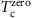
=18.0 K, which is ∼30% higher than that found in the bulk materials^32^. Strikingly, we found that the irradiated FST film does not show any *T*_c_ degradation, rather an increase of *T*_c_ by ∼0.5 K. The enhancement of *T*_c_ by irradiations, with either light ion or heavy ion, was not known in iron-based superconductors previously^27^,^28^,^29^,^30^,^31^.

In iron-chalcogenide superconductors, there have been many reports on enhancement of *T*_c_ for bulk materials^33^,^34^,^35^,^36^,^37^, wires^38^ and films^16^,^39^,^40^,^41^,^42^,^43^,^44^. Possible explanations can be grouped into three categories so far: (1) an interface effect, (2) an elimination of the influence of excess Fe at the interstitial sites of the Te/Se(S) layer and (3) a strain effect.

The first group is based on the observation of monolayer FeSe films on SrTiO_3_ substrate^39^,^40^. The enhanced superconductivity is generally believed to be the result of the interface between two very different materials and not relevant to our FST film of ∼100 nm thick.

**Figure 2:**
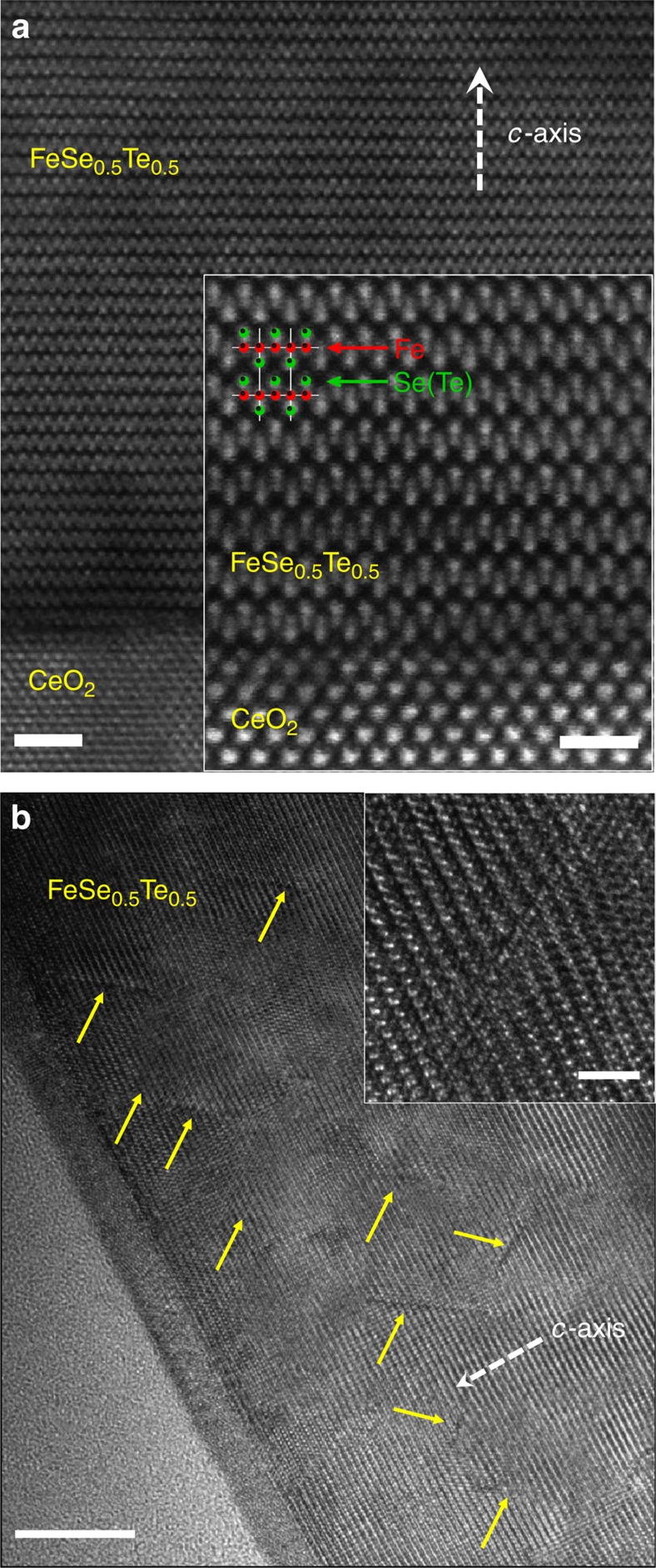
Microstructure of FST films. (**a**) STEM-HAADF images of the representative FST film on the CeO_2_ buffer layer. Scale bar, 2 nm. Inset: high-resolution image at the interface between CeO_2_ and FST. Scale bar, 1 nm. (**b**) HRTEM image of FST film irradiated with 190 keV proton. Scale bar, 10 nm. Inset: high-resolution image of splayed cascade defect and strain field produced by 190 keV proton irradiation. Scale bar, 2 nm.

The second group is based on the post-annealing effect^35^,^36^,^37^. This could be attributed to a reduction of excess Fe, which creates magnetic moments and causes pair breaking^45^. The reasonable concern could be raised that the enhancement of *T*_c_ in the proton-irradiated FST films is due to the suppression of the influence of excess Fe. To address this concern, we performed extensive high-resolution TEM (HRTEM) and scanning TEM (STEM) with high-angle annular dark field detector (HAADF) characterization. Figure 2a displays representative STEM–HAADF image of the pristine FST film. The FST film is epitaxially grown on the CeO_2_ buffer layer. STEM–HAADF image also reveals the very sharp FST-CeO_2_ interface without the chemical reaction layer reported in FST/CaF_2_ interface^42^. What is more remarkable here is that excess Fe, which is supposed to occupy the interstitial site in many iron-chalcogenide bulk samples, is not present in the FST film. Note: the presence of excess Fe in the interstitial sites is one of the major issues in iron chalcogenide superconductor, which lowers *H*_c2_. The absence of excess Fe in our FST films deposited by pulsed laser deposition method is consistent with the observation that these FST films retain excellent performance at high field^15^,^16^,^46^. This is of great advantage for high-field applications.

The third group is related to the strain effect on *T*_c_ (refs 33, 34, 38), especially in iron-chalcogenide superconducting films^16^,^41^,^42^,^43^,^44^. Figure 2b shows a cross-sectional HRTEM image of the FST film irradiated with 190 keV proton. We observed splayed cascade defects produced by proton irradiation over the entire film. An enlarged view of a typical defect is displayed in the inset. The stopping range of ions in matter model^47^ predicts that with increasing depth in Al foil covered on the FST film (Supplementary Fig. 1), the angle distribution of the proton broadens, producing an angular splay for the proton. Previously, disordered spherical cascade defects were found to exist in the low-energy region of the Bi_2_Sr_2_CaCu_2_O_*x*_ crystals^22^. Given that nuclear scattering events produce angular deviations from the original incident angle as the incident ions proceed through a target material, it is not surprising that light mass proton with a low energy of 190 keV easily tilted away from the original incident angle (0°) and create the disordered cascade defects.

According to Anderson's theorem^48^, the *T*_c_ and the superconducting density of states in conventional *s*-wave superconductors are not affected by the non-magnetic impurity created by proton irradiation, whereas non-magnetic impurities can act as strong scattering centres and suppress *T*_c_ by pair breaking in *d*-wave^49^ or *s*± wave^50^ superconductors. In striking contrast, here we observed enhanced *T*_c_ in the FST films after proton irradiation. Now we propose another mechanism to explain the enhancement of *T*_c_ by proton irradiation. We note that besides the clearly visible cascade, there is also a curvature of the lattice fringe inwardly directed, which produces strain field around the cascade defect. Similar strain fields created by low-energy proton and neutron irradiation were observed in YBCO single crystals from a previous TEM study^51^,^52^. Although the size of the cascade defects is about 1∼2 nm in diameter and ∼10 nm wide, the strain fields are expected to extend far out to the cascade defect, especially in the radial direction and, as a result, can cause the local lattice distortion.

### Origin of enhanced *T*_c_ and nanoscale lattice strain

**Figure 3:**
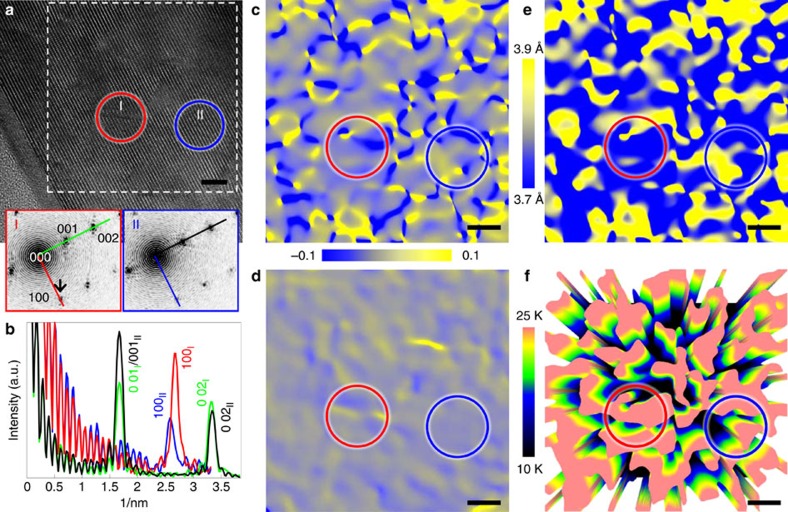
Strain analysis in a 190 keV proton irradiated FST film. (**a**) HRTEM image. The inset I and II are the diffractogram from the circled area I (red) and II (blue), respectively. (**b**) Intensity line profiles of 001/002 (green and black) and 100 (red and blue) spots from the scan lines shown in the inset I and II of **a**. (**c**,**d**) Strain maps of in-plane *ɛ*_*xx*_ (**c**) and out-of-plane *ɛ*_*zz*_ (**d**) calculated by geometrical phase analysis (GPA)^53^,^54^,^55^ from the square area outlined by dash line in **a**. The colour bar in the middle indicates the strain from −0.1 (compressive) to 0.1 (tensile). (**e**) Map of in-plane lattice parameter determined from the GPA map in **c**. The colour bar in the left indicates the change in lattice parameter from 3.7 to 3.9 Å. (**f**) Perspective view of the spatial distribution of *T*_c_ calculated from the local strain (Supplementary Fig. 3). The red and blue circles show the same area. Scale bar, 5 nm (**a**,**c**–**f**).

To see the cascade defect induced local lattice distortion, we Fourier transform two circled areas I and II in Fig. 3a, to get the diffractogram of the area of interest, as shown in the inset I and II. Intensity profiles from the central spot (000) to the reflection spots is then obtained for the measurement and the comparison of the positions of the reflection spots, as shown in Fig. 3b. The position of the 002 spot from circle I (green line) just slightly shift to the left in comparison with that from the circle II (black line), indicative of almost same *c*-lattice parameter in the irradiated FST film. On the other hand, the position of the 100 peak from local area I and II are not the same, indicative of large lattice variation along *a* axis parameter. We can calculate the strain map by geometrical phase analysis^53^,^54^,^55^. Figure 3c,d show the in-plane *ɛ*_*xx*_ (*a* axis direction) and the out-of-plane *ɛ*_*zz*_ (*c* axis direction) maps retrieved from the square area marked by the dash line in Fig. 3a. The averaged *a*- and *c*-lattice parameters were determined to be 3.77 and 5.95 Å, respectively, from the diffractogram of the whole image. The map of *ɛ*_*xx*_ (Fig. 3c) shows extreme spatial variation of strains. Deep valleys (blue) are the highly compressed region with compressive strain *ɛ*_*xx*_≅−0.1, whereas sharp peaks (yellow) are the highly stretched region with tensile strain *ɛ*_*xx*_≅0.1. These two extreme domains are nanoscopically entangled with each other in the irradiated FST film. This is in striking contrast to the map of *ɛ*_*zz*_ in Fig. 3d, which is dominated by large interconnected areas of weak, strained areas. In contrast to the large strain observed in the irradiated FST film, the unirradiated FST film shows little strain in both in-plane *ɛ*_*xx*_ and out-of-plane *ɛ*_*zz*_ (Supplementary Fig. 2). From the *ɛ*_*xx*_ in the irradiated FST film, we can calculate lattice parameter map: *a*=*a*_0_(1+*ɛ*_*xx*_), where *a*_0_ is the lattice parameter from the reference area (solid blue circle in Fig. 3a). The result is shown in Fig. 3e. We noticed that low-energy proton irradiation causes more than 5% variation in lattice parameter *a* ranging from 3.7 to 3.9 Å over merely a few nanometre scale randomly distributed in the FST films. Red circles in Fig. 3a–f show the same area containing one cascade defect. Under electron, neutron or ion irradiation, point defect clusters are also produced in the form of vacancy and interstitial clusters from collision cascade defects in irradiated materials^20^,^21^,^51^,^52^. These clusters could cause the great compression-–tension strain fields at a nanoscale alongside the cascade defects.

It is well known that compressive strain, especially along the in-plane direction, plays a crucial role in enhancing *T*_c_ in FST films^16^,^41^,^42^,^43^. Now, we attempt to correlate the strain and *T*_c_ in the irradiated films quantitatively, based on the analysis given by Bellingeri *et al*.^41^, that *a* axis lattice parameter is approximately linearly proportional to *T*_c_. A three-dimensional false-colour image in Fig. 3f shows the spatial variation of *T*_c_ obtained by using the linear relation given by Bellingeri *et al*.^41^ (Supplementary Fig. 3). High-*T*_c_ (highly compressive) and low-*T*_c_ (highly tensile) regions are linked in a network similar to a cobweb in the irradiated FST films, with the highest *T*_c_ reaching ∼25 K and the lowest *T*_c_ reaching ∼15 K. The enhanced *T*_c_ regions in the irradiated FST film rises from these contracted lattice parameter domains (highly compressive strain region) that are just 5 nm apart in average. This distance is comparable to the superconducting coherence length of FeSe_1–*x*_Te_*x*_(0.5<*x*<0.85), estimated to be around 2.6 nm at zero temperature using the Ginzburg–Landau theory^56^,^57^. The irradiated FST films consist of small puddles of different *T*_c_ values as shown in Fig. 3f. Proximity effect should lead to near homogeneous *T*_c_ (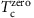
∼18 K) in the film as a whole. There is greater volume associated with the compressive strain in *a* axis direction than that with tensile strain in the irradiated FST films, resulting in the higher value of *T*_c_ as demonstrated in Fig. 1.

### Irreversibility field and upper critical field

**Figure 4:**
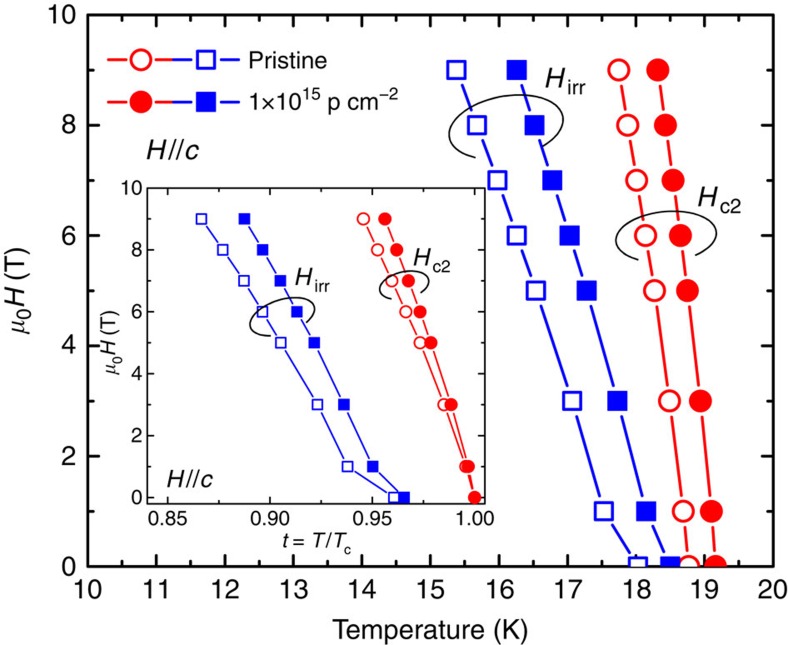
Upper critical field and irreversibility field. Upper critical field *H*_c2_(*T*) and irreversible field *H*_irr_(*T*) (*H*//*c*) as a function of temperature up to 9 T//*c* for FST films before and after proton irradiation determined from Fig. 1. Inset: normalized temperature (*t*=*T/T_c_*) dependence of *H*_c2_ and *H*_irr_ for the same field orientation. Error bars are of a size smaller than the data points.

Now, we turn our attention to vortex pinning behaviour in the irradiated FST films. Figure 4 shows the temperature dependence of irreversibility field *H*_irr_ and *H*_c2_ for *H*//*c*. Both *H*_irr_ and *H*_c2_ shift to higher *T* for the proton-irradiated film. When plotted as a function of *t*=*T*/*T*_c_, *H*_irr_ and *H*_c2_ increase after proton irradiation can be compared. We found the increase in *H*_irr_ is even more significant and produces a larger pinned vortex region, which suggests the cascade defects are strong pinning centres^20^,^21^,^58^. This is particularly important for the potential applications of the iron-based superconductor, as it can be operated in a larger-phase space.

### *J*_c_ in high magnetic field up to 34.5 T

**Figure 5:**
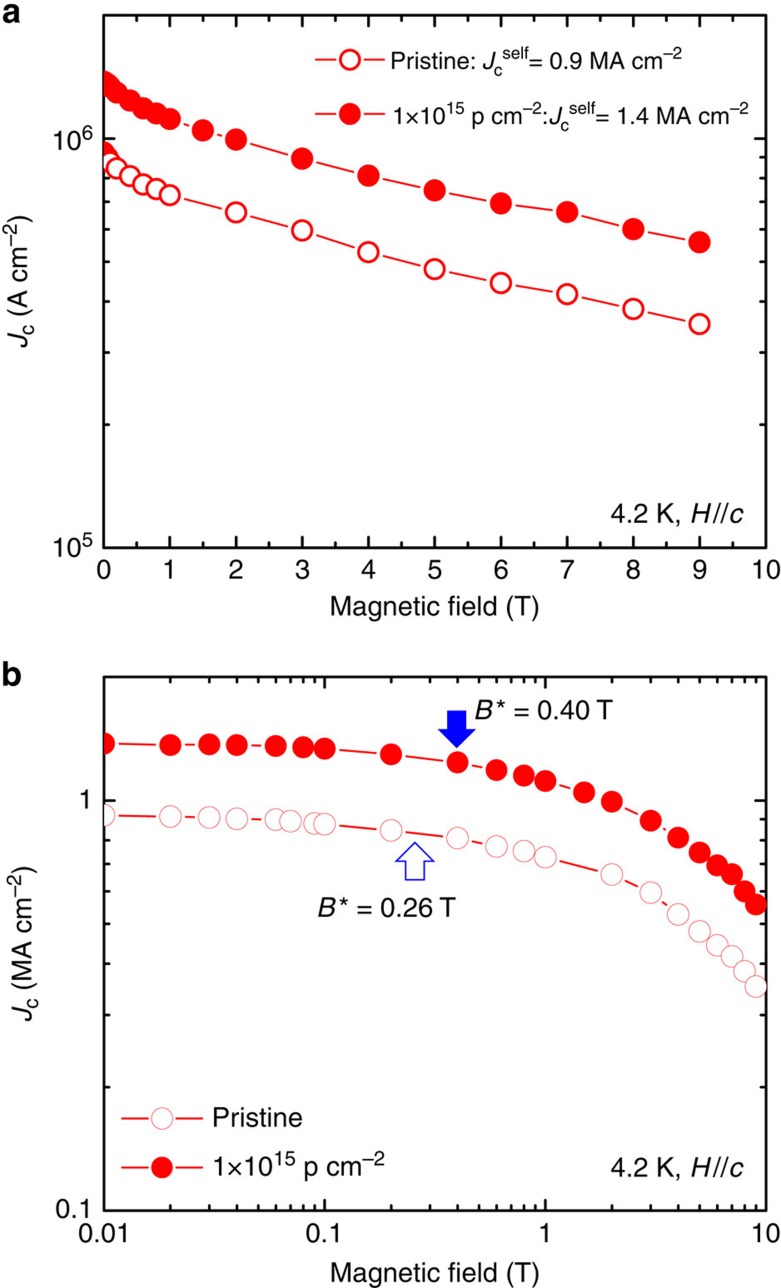
Critical current density as a function of magnetic fields up to 9 T. (**a**) *J*_c_(*H*//*c*) for the FST film before and after 190 keV proton irradiation with 1 × 10^15^ p cm^−2^ dose at 4.2 K. (**b**) Same data plotted in log-log scale. The arrows indicate the field of the crossover field B*.

Strong enhancement of *J*_c_ is shown in Fig. 5a, where we plot *J*_c_(*H*) for *H*//*c* at 4.2 K for 190 keV proton irradiation. The irradiated FST film has a larger self- and in-field *J*_c_ than the pristine film. The self-field *J*_c_ enhances from 0.9 MA cm^–2^ up to 1.4 MA cm^–2^. Normalized *J*_c_(*H*) dependence of the FST films before and after irradiation is shown in a log–log pot in Fig. 5b. The regime of a nearly constant *J*_c_ up to a characteristic crossover field of B* (90% of normalized *J*_c_(*H*)), is associated with a single vortex pinning regime, followed by a rollover to a power-law regime (*J*_c_∝*B*^–*α*^) at intermediate fields, at which vortex–vortex interactions become relevant and a collective pinning vortex motion is dominant. We found that the pristine FST film already has high B* of 0.26 T, which is higher than BaFe_2_As_2_:P+BaZrO_3_ films^8^. After proton irradiation, B* in the FST film increases up to 0.40 T. Studies by Dam *et al*.^59^ showed that no correlation is found between the density of linear defects and the value of *J*_c_ in the single vortex-pinning regime. The ratio between B*'s before and after irradiation is almost the same as the one between self-field *J*_c_'s. Thus, the increase in B* after irradiation might just come from the increase in the self-field *J*_c_. At intermediate field, however, *J*_c_(*H*) in both FST films does not follow a power-law decay, although we observe a roundish *J*_c_ dependence with magnetic field, as shown in Fig. 5b. The similar behaviour, that is, the absence of a power-law decay (or almost no change in *α*), has been found in both cuprate and iron-based superconducting films with strong vortex pinning^6^,^7^,^8^,^60^. It appears the irradiated FST films also exhibit an extremely good *J*_c_ retention at low field, indicating the potential of the iron-based superconductors for utility cable application.

**Figure 6:**
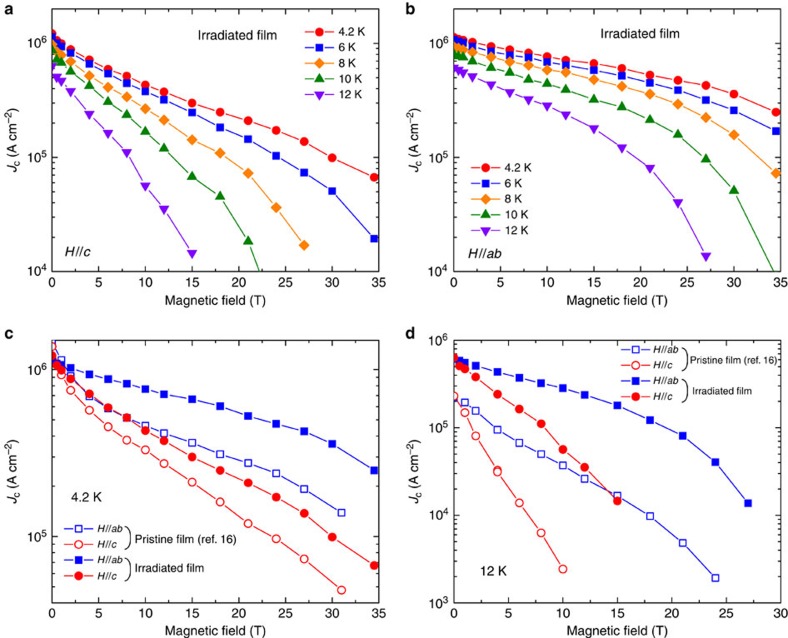
High-field critical current density up to 34.5 T. (**a**,**b**) *J*_c_(*H*) for proton-irradiated FST film with 1 × 10^15^ p cm^−2^ dose at different temperature up to 34.5 T with *H*//*c* and *H*//*ab*, respectively. (**c**,**d**) *J*_c_(*H*) for the irradiated FST film up to 34.5 T compared with the pristine FST film^16^ at 4.2 and 12 K, respectively.

High-magnetic field transport measurement of the irradiated FST film was performed up to 34.5 T. Figure 6a,b show *J*_c_(*H*) at various temperatures for *H*//*c* and *H*//*ab*, respectively. In Fig. 6c,d, we plot *J*_c_(*H*) for the irradiated FST film at 4.2 and 12 K, together with the *J*_c_(*H*) data taken on the pristine film^16^ for comparison. The irradiated FST film clearly has a much better field performance for both *H*//*ab* and *H*//*c*. We found the *J*_c_ at 12 K increased by one order of magnitude after irradiation over 15 T for *H*//*ab* and over 6 T for *H*//*c*. Enhancement of vortex pinning at higher temperature (12 K) is much more significant compared with that at low temperature (4.2 K).

### Vortex-pinning properties

**Figure 7:**
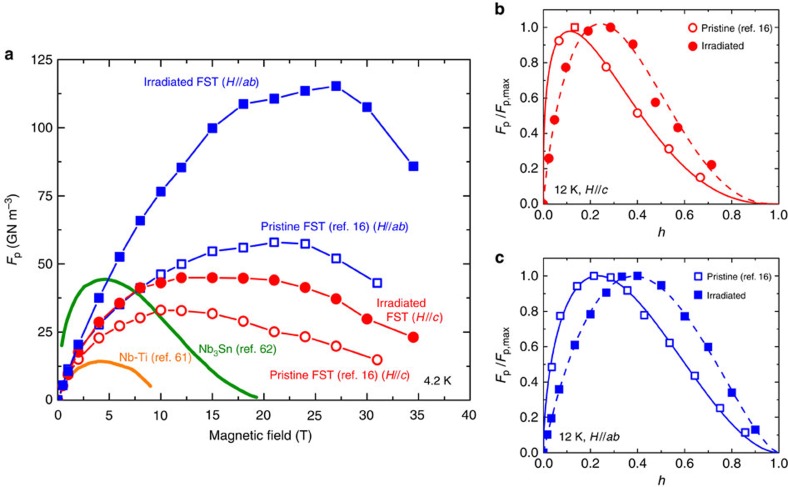
High-field pinning force. (**a**) *F*_p_(*H*) for FST film before and after 190 keV proton irradiation with 1 × 10^15^ p cm^−2^ dose at 4.2 K, compared with NbTi (ref. 61) and Nb_3_Sn (ref. 62) wires. (**b**,**c**) Normalized *F*_p_(*h*) for FST film before (open symbols) and after (close symbols) proton irradiation at 12 K for *H*//*c* and *H*//*ab*, respectively, whereas solid (before) and dashed (after) lines are the corresponding fitting curves.

The effect of cascade defects and strain modulation from proton irradiation on vortex pinning properties can be appreciated by evaluating the pinning force *F*_p_=*J*_c_ × *μ*_0_*H*. Figure 7a shows *F*_p_(*H*) for the pristine^16^ and the irradiated FST films at 4.2 K together with the data for NbTi (ref. 61) and Nb_3_Sn (ref. 62) wires for comparison. After proton irradiation, the *F*_p_ peak width broadens and the enhancement of *F*_p_ is prominent in the high-field region for *H*//*c*. The maximum pinning force *F*_p,max_∼45 GN m^–3^, which is increased by ∼30%, occurs at 12 T in the irradiated FST film for *H*//*c*. At *H*//*ab*, a remarkably high *F*_p,max_ of ∼120 GN m^–3^ is reached for the irradiated FST film at 27 T, that is, an enhancement of ∼200% over the pristine film. These results suggest that the cascade defects and nanoscale strain field can be highly effective pinning centres, especially for *H*//*ab*. Extensive researches by Llordés *et al*.^63^ and Deutscher^64^ have showed a similar result that the nanoscale strain present in *ɛ*_*zz*_ map is responsible for a strong enhancement of pinning force for *H*//*c*. In the irradiated FST film, the nanoscale strain observed in *ɛ*_*xx*_ map can lead to the extremely high *F*_p,max_ and the shift of the *F*_p,max_ to high field for *H*//*ab*.

The reduced pinning force density 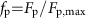 as a function of reduced magnetic field 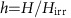 for the pristine^16^ and the irradiated FST films at 12 K is plotted in Fig. 7b,c, respectively. Solid and dashed fitting lines are calculated using 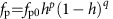, where *f*_p0_ is a constant. The values of exponent fitting parameters *p* and *q* are given in the Supplementary Table 1. After irradiation, we found that the exponent *p* increases significantly to 0.57 for *H*//*c* from 0.33 before the irradiation, whereas for *H*//*ab*, *p* increases to 0.97 (towards unity) from 0.85 before the irradiation. The exponent *q* decreases substantially to 1.87 (*H//c*) and 1.56 (*H*//*ab*) from the values of 2.63 (*H*//*c*) and 2.68 (*H*//*ab*) before the irradiation. It is perhaps instructive to compare our results with the vortex pinning for *α*-Ti ribbon in Nb-Ti wires, which shows that cold work reduces the dimensions of the *α*-Ti precipitates to optimum size for vortex pinning, resulting in the *F*_p_ curve shape 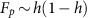 (refs 65, 66). Of course, rigorous analysis and modelling would need to take into account of accurate information on the defect size, associated strain field and morphology/anisotropy, which is beyond the current studies. Nevertheless, both fitting parameters *p* and *q* are found approaching unity in the irradiated films, which is consistent with the structural observation shown in Fig. 3. The cascade defect and nanoscale strain act more or less similar to elongated point-defect pinning. The scaling behaviour 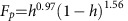 for *H*//*ab* of the irradiated FST film may be attributed to strong vortex pinning due to a dense array of nanoscale cascade defect and longer extension of the strain field along the *a* axis direction.

In conclusion, a robust enhancement of *T*_c_ and *J*_c_ has been realized simultaneously in the FST film irradiated with 1 × 10^15^ p cm^–2^ dose of 190 keV proton, resulting in an increase of 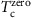 from 18.0 to 18.5 K and a self-field *J*_c_ enhancement from 0.9 MA cm^–2^ up to 1.4 MA cm^–2^ at 4.2 K. TEM structural characterization provides direct atomic-scale imaging of the collision cascade defects and the surrounding strain field, produced by low-energy proton irradiation. The highly compressive strain increases *T*_c_ of the irradiated films through the proximity effect. The cascade defects and nanoscale strain are found to provide strong vortex pinning, leading to the increase of *J*_c_ by one order of magnitude at 12 K over 6 T and record high *F*_p,max_ of ∼120 GN m^–3^ for *H*//*ab* at 27 T and 4.2 K. This study demonstrates that it is possible to achieve significant enhancement of *J*_c_ without *T*_c_ reduction through the design of vortex pinning landscape that takes advantage of cascade defects and associated strain field. Further improvement of the performance of these films can be expected with fine tuning of the morphology of defects and strain configuration. The low-energy ion sources are inexpensive to operate and are readily available commercially. This route provides a practical solution to the central limiting factor that controls the performance of superconducting wires and tapes that underpins all superconducting applications.

## Methods

### Sample preparation

FST films of 100–130 nm thickness are grown by pulsed laser deposition with CeO_2_ buffer layer on SrTiO_3_ single-crystalline substrates^15^,^16^,^46^. The FST films characterized in advance were covered by 1.5 μm thick Al foil and irradiated with 190 keV protons at dose of 1 × 10^15^ p cm^–2^ (Supplementary Fig. 1). At this energy, the simulation using the stopping range of ions in matter code^47^ shows that the stopping region (Bragg peak) of the protons is ∼100 nm from the FST film surface, which maximizes the effect of cascade defect creations in the FST films.

### Characterization

Microstructures of the 190 keV proton-irradiated FST film were characterized using aberration-corrected STEM and HRTEM.

All transport characterizations were performed on the same films before and after irradiation. Low-field measurements were performed by the standard four-probe method in a physical property measurement system (Quantum Design), whereas the high-field measurements were done in 35 T direct current magnet at National High Magnetic Field Laboratory in Tallahassee.

### Data availability

All relevant data supporting the finding of this study are available within the article and its Supplementary Information file or available from the corresponding authors upon request, which should be addressed to T.O. and Q.L.

## Additional information

**How to cite this article:** Ozaki, T. *et al*. A route for a strong increase of critical current in nanostrained iron-based superconductors. *Nat. Commun.* **7,** 13036 doi: 10.1038/ncomms13036 (2016).

## Supplementary Material

Supplementary Information Supplementary Figures 1-3, Supplementary Table 1 and Supplementary References
