# Supplementary material for: A route for a strong increase of critical current in nanostrained iron-based superconductors
Source: Nat Commun. 2016 Oct 6;7:13036. doi: 10.1038/ncomms13036 (PMC5059717; doi:10.1038/ncomms13036)
Supplement: Supplementary Information — Supplementary Figures 1-3, Supplementary Table 1 and Supplementary References [file ncomms13036-s1.pdf]

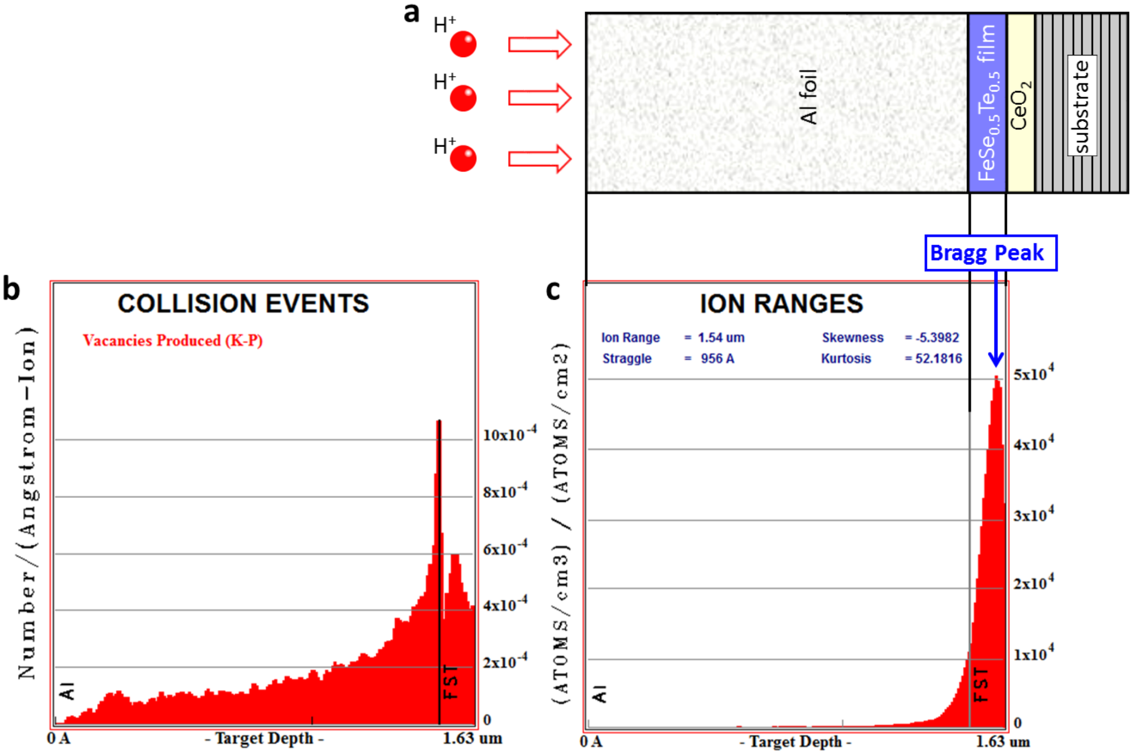

Supplementary Figure 1. **Simulation results using SRIM (stopping range of ions in matter) code[1]. a** the configuration of the $FeSe_{0.5}Te_{0.5}$ (FST) films covered by the Al foil. **b,c** Example of the ion range (b) and collision events (c) as a function of target depth produced from SRIM calculation for 190 keV proton irradiation of 130 nm thick FST superconducting films. We use SRIM code for the design of low-energy proton irradiation of our FST films. To maximize the efficiency of pinning defects creation, a 1.5 μm thick Al foil is placed on the top of the 130 nm thick FST film to have the stopping range (Bragg peak) inside the FST films. Bragg peak is around 100 nm from the top of the FST film surface. In this article, the FST films were irradiated with 190 keV protons at dose of 1×10^{15} p cm^{-2}. The total number of the displacement per unit volume is $\sim5.0\times10^{19}$ cm^{-3}, leading to the mean distance between the introduced defects of ~3 nm and the amount of displacement damage of $\sim1.1\times10^{-3}$ dpa (displacement per atom).

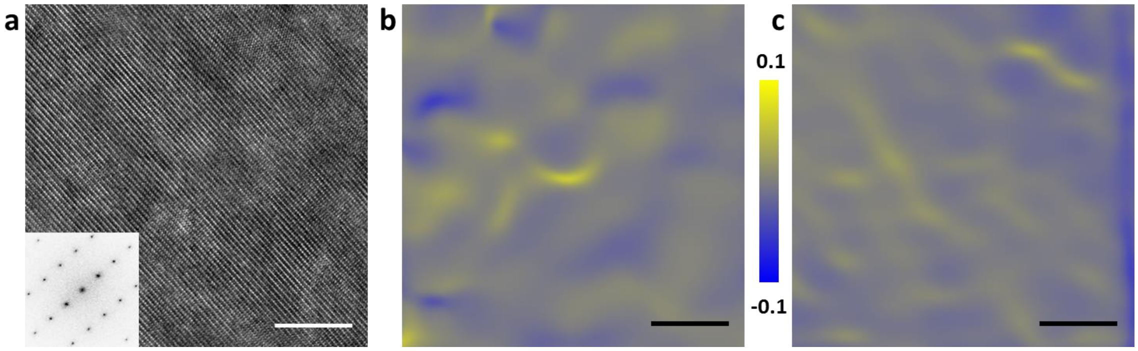

Supplementary Figure 2. **Strain analysis in the $FeSe_{0.5}Te_{0.5}$ films prior to irradiation.** **a** HRTEM image. The inset is the FFT of the whole image. **b,c** Strain maps of in-plane ε_{xx} (b) and out-of-plane ε_{zz} (c) calculated by geometrical phase analysis (GPA)[2-4]. The color bar in the middle indicates the strain from –0.1 (compressive) to 0.1 (tensile). Scale bar, 10 nm (a-c). For a direct comparison of the microstructures of the $FeSe_{0.5}Te_{0.5}$ (FST) superconducting films before and after proton irradiation, we also performed structural characterization and analysis of an unirradiated film prepared at the same time as the irradiated one during the PLD. The strain contrast in the pristine films is weak, and the strain variation is over the scale of tens of nm, which is very different from the intense strain variation over the scale of just a few nm in the irradiated films, as shown in Fig. 3.

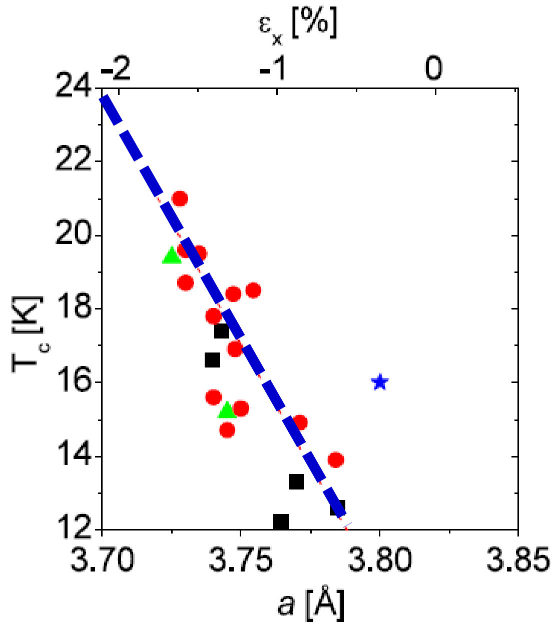

Supplementary Figure 3. **The critical temperature T_c as a function of *a* lattice parameter.** The critical temperature T_c of several FST films on $LaAlO_3$ (circles), $SrTiO_3$ (squares) and Y:ZrO (triangles) are plotted as a function of the in-plane *a* lattice parameter. The star represents the bulk value – all data are adopted from the original figure 7 of reference #5. The line is the least square fit to the data for the films. To map the local T_c of the irradiated film (Fig. 3f), we use the analysis given by E. Bellingeri *et al.*[5] that *a*-axis lattice parameter is approximately linear-proportional to T_c. We obtained the T_c and the lattice parameter *a* relation: T_c [K] ≅ –131.5×*a* [Å] + 510.3.

Supplementary Table 1. **Fitting parameters p and q in the reduced pinning force density at 12 K.** The reduced pinning force density $f_\mathrm{p} = F_\mathrm{p}/F_\mathrm{p,max}$ as a function of reduced magnetic field $h = H/H_\mathrm{irr}$ for the pristine and the irradiated FST films at 12 K is plotted in Fig. 7b,c, respectively. Solid and dashed fitting lines are calculated using $f_\mathrm{p} = f_\mathrm{p0} h^p (1-h)^q$, where f_p0 is a constant.

Sample	*p*	*q*
Pristine FST[6], *H*//*c*	0.33±0.05	2.63±0.17
Pristine FST[6], *H*//*ab*	0.85±0.07	2.68±0.20
Irradiated FST, *H*//*c*	0.57±0.03	1.87±0.08
Irradiated FST, *H*//*ab*	0.97±0.02	1.56±0.03

Supplementary References

1) Ziegler, J. F., Biersack, J. P. & Littmark, U. The Stopping and Range of Ions in Solids (Pergamon, New York, 1985).
2) Hÿtch, M. J., Putaux, J.-L. & Pénisson, J.-M. Measurement of the displacement field of dislocations to 0.03 Å by electron microscopy. Nature **423**, 270-273 (2003).
3) Hÿtch, M. J., Snoeck, E. &Kilaas, R. Quantitative measurement of displacement and strain fields from HREM micrographs. Ultramicroscopy **74**, 131-146 (1998).
4) Wu, L. *et al*. Nanostructures and defects in thermoelectric $AgPb_{18}SbTe_{20}$ single crystal. *J. Appl. Phys.* **105**, 094317 (2009).
5) Bellingeri, E. *et al.* Critical temperature enhancement by biaxial compressive Strain in $FeSe_{0.5}Te_{0.5}$ Thin Films. *J. Supercond. Nov. Magn.* **24**, 35−41 (2011).
6) Si, W. *et al.* High current superconductivity in $FeSe_{0.5}Te_{0.5}$-coated conductors at 30 tesla. *Nat. Commun.* **4**, 1347 (2013).
